# Premature ovarian failure

**DOI:** 10.1186/1750-1172-1-9

**Published:** 2006-04-06

**Authors:** Paolo Beck-Peccoz, Luca Persani

**Affiliations:** 1 Dipartimento di Scienze Mediche, Università di Milano, IRCCS Fondazione Ospedale Maggiore Policlinico, Mangiagalli e Regina Elena, Via F. Sforza 35, 20122-Milano, Italia; 2 Dipartimento di Scienze Mediche, Università di Milano, IRCCS Istituto Auxologico Italiano, Via Zucchi 18, 20095 Cusano (Milano), Italia

## Abstract

Premature ovarian failure (POF) is a primary ovarian defect characterized by absent menarche (primary amenorrhea) or premature depletion of ovarian follicles before the age of 40 years (secondary amenorrhea). It is a heterogeneous disorder affecting approximately 1% of women <40 years, 1:10,000 women by age 20 and 1:1,000 women by age 30. The most severe forms present with absent pubertal development and primary amenorrhea (50% of these cases due to ovarian dysgenesis), whereas forms with post-pubertal onset are characterized by disappearance of menstrual cycles (secondary amenorrhea) associated with premature follicular depletion. As in the case of physiological menopause, POF presents by typical manifestations of climacterium: infertility associated with palpitations, heat intolerance, flushes, anxiety, depression, fatigue. POF is biochemically characterized by low levels of gonadal hormones (estrogens and inhibins) and high levels of gonadotropins (LH and FSH) (hypergonadotropic amenorrhea). Beyond infertility, hormone defects may cause severe neurological, metabolic or cardiovascular consequences and lead to the early onset of osteoporosis. Heterogeneity of POF is also reflected by the variety of possible causes, including autoimmunity, toxics, drugs, as well as genetic defects. POF has a strong genetic component. X chromosome abnormalities (*e.g. *Turner syndrome) represent the major cause of primary amenorrhea associated with ovarian dysgenesis. Despite the description of several candidate genes, the cause of POF remains undetermined in the vast majority of the cases. Management includes substitution of the hormone defect by estrogen/progestin preparations. The only solution presently available for the fertility defect in women with absent follicular reserve is ovum donation.

## Disease name and synonyms

Premature ovarian failure (POF; POF1: OMIM 311360); Hypergonadotropic ovarian failure; Menopausa precoce.

### Included diseases

POF2 (OMIM #300511); POF3 (OMIM #608996)

## Definition

Premature ovarian failure is defined as a primary ovarian defect characterized by absent menarche (primary amenorrhea) or premature depletion of ovarian follicles/arrested folliculogenisis before the age of 40 years (secondary amenorrhea) [1,2].

## Epidemiology

POF affects approximately: one in 10,000 women by age 20; one in 1,000 women by age 30; one in 100 women by age 40 [3]. The familial form of POF is rare, representing 4 to 31% of all cases of POF [4-6].

## Etiology

The causes of POF are extremely heterogeneous. Acquired forms such as those occurring after treatments for neoplastic diseases or autoimmune diseases account for many cases [1]. POF has a strong genetic component with X chromosome abnormalities playing a primary role mainly in the cases with ovarian dysgenesis [7-10]. A gene (or genes) for POF (POF1) was localised to Xq21.3–Xq27 on the basis of deletions in various patients and families. A second gene (or genes) for POF (POF2) implicated by the analysis of balanced X/autosomal translocations has been localised to Xq13.3–q21.1. Despite the description of several candidate genes [11-16], the cause of POF still remains undetermined in the majority of the cases (idiopathic). This idiopathic form of POF can show sporadic and familial forms.

The different causes of POF are illustrated as follows:

• Iatrogenic origin (surgery, chemotherapy, radiations);

• Autoimmune, including polyglandular autoimmune syndrome, as well as autoimmune polyendocrinopathy-candidiasis-ectodermal dystrophy (APECED) due to mutations in *AIRE *gene);

• Infections (*e.g. *herpes zoster, cytomegalovirus);

• Chromosome X defects:

■ Turner syndrome

■ Fragile X syndrome (*FMR1 *gene premutation)

• Monogenic defects

■ Syndromic defects:

○ Congenital disorders of glycosylation (CDG, formerly named carbohydrate-deficient glycoprotein syndromes) (recessive)

○ Galactosemia (recessive)

○ Blepharophimosis-ptosis-epicanthus inversus syndrome (BPES) (female-limited, dominant)

○ Pseudohypoparathyroidism (PHP) type Ia (parental imprinting : maternal inheritance)

■ Isolated defects:

○ Follicle stimulating hormone (FSH) receptor mutations (*FSHR*), (recessive)

○ Luteinizing hormone (LH) receptor mutations (*LHR*), (recessive)

○ *FOXL2 *(transcription factor involved in BPES) mutations (female-limited defect, dominant)

○ Bone morphogenetic protein 15 (*BMP15*) mutations (female-limited defect, heterozygous mutation)

• Idiopathic

Defects in some of these candidate genes may present with different phenotypes. *FOXL2 *defects may present either with BPES type 1 (without POF) or with BPES type 2 (with POF), condition designated as POF3 [14]. Rarely, *FOXL2 *mutations may be associated with POF in the absence of eyelid/palpebral alterations (isolated POF) [17,18]. Depending on the degree of FSH resistance, *FSHR *defects are associated with primary [12] or secondary amenorrhea [13,19]. Mutations in *LHR *have been described in women with secondary amenorrhea (characterized by elevated serum LH/FSH ratio and cystic follicles at ultrasound) belonging to pedigrees of male patients with Leydig hypoplasia [20]. Two of the candidate genes are located on the X chromosome. *FMR1 *gene (Xq27.3) mutations or pre-mutations are typically associated with secondary amenorrhea in female relatives of male patients with mental retardation [8]. *BMP15 *gene (Xp11.2) defect has so far been described in two sisters with primary amenorrhea and heterozygous for the mutation. This defect represents an unusual example of a X-linked disease in which affected females inherit the mutation from their unaffected father [15].

## Clinical description

The symptoms can vary considerably from patient to patient and the disorder may occur abruptly or spontaneously or it may develop gradually over several years. The most severe forms of hypergonadotropic ovarian failure present with absent pubertal development and primary amenorrhea [2,21]. The clinical picture is characterized by absent menarche and pubertal delay results in absent sexual maturation and reduced growth velocity. In the female, pubertal delay is defined as the absence of mammary and pubic hair development and menarche at 13 years. Moderate hirsutism may be seen due to the action of androgens originating from adrenals.

About half of the cases of primary amenorrhea are due to ovarian dysgenesis, which is revealed by the finding of streak ovaries accompanied by uterus hypoplasia at ultrasound. In the other patients, follicles (<10 mm) may be found at histological evaluation such as in the case of *FSHR *mutations [22]. In these cases, almost normal pubertal development may be seen.

Post-pubertal onset of ovarian failure represents the large majority of the cases [1]. This is characterized by secondary amenorrhea associated with premature follicular depletion or arrested folliculogenisis. As in the case of physiological menopause, POF is clinically characterized by typical manifestations of climacterium such as palpitations, heat intolerance, flushes, night sweats, irritability, anxiety, depression, sleep disturbance, decreased libido, hair coarseness, vaginal dryness, fatigue.

Female infertility is an obvious and presently irreversible consequence of POF. Importantly, POF and prolonged lack of estrogen treatment may lead to the early onset of osteopenia and osteoporosis. Moreover, sexual hormone defects represent an important risk factor for frequent and severe neurological, metabolic or cardiovascular disorders such as Alzheimer's disease, hypercholesterolemia or ischemic diseases.

Hypergonadotropic ovarian failure may be part of other syndromic features (see the causes of POF): Autoimmune polyendocrinopathy-candidiasis-ectodermal dystrophy, Blepharophimosis-ptosis-epicanthus inversus syndrome, Carbohydrate-deficient glycoprotein syndromes, Galactosemia, Turner) and PHP I.

PHP I is associated with mutation in the *GNAS *gene encoding Gs protein alpha [23]. The diagnosis of PHP I is based on the findings of resistance to several peptide hormones acting through the adenylyl cyclase/cAMP pathway. The key findings are elevated parathyroid hormone (PTH) with low/normal calcemia, high thyrotropin (TSH) with normal thyroid hormone levels, growth hormone deficiency and high gonadotropins in patient with delayed puberty and skeletal abnormalities (Albright osteodystrophy). PHP I syndrome occurs when the mutant allele is inherited from the mother, due to the imprinting of the paternal *GNAS *allele in the affected tissues.

The early diagnosis of familial POF will provide the opportunity to predict the likelihood of early menopause, and allow other reproductive choices to be made, such as freezing embryos or having children earlier. As POF has cumulative negative effects over time, it is important for clinicians to make a timely diagnosis and begin appropriate strategies for symptom management, emotional support, and risk reduction.

## Diagnostic methods

Both primary and secondary forms of ovarian failure are biochemically characterized by low levels of gonadal hormones (estrogens and inhibins) and high gonadotropins (LH and FSH) (hypergonadotropic amenorrhea). The elevation of FSH is usually more marked than that of LH and an FSH value >30 U/L is indicative of ovarian failure.

Ultrasound frequently reveals small ovaries without evidence of growing follicles. In the cases with primary amenorrhea, gonadal dysgenesis is documented by the finding of streak ovaries. Histological examination of biopsies performed during pelvic laparoscopy in the case of hypoplastic ovaries (0.20–0.30 ml on ultrasound) may reveal the presence of primary follicles. Forms of POF linked to the finding of ovarian cysts may be due to LH resistance (*LHR *mutations) which presents with secondary amenorrhea. In contrast to what is generally found in POF, defects in LH receptor are typically associated with a serum LH elevation (> 10 U/L) more pronounced than that of serum FSH. The evaluation of other peptide factors of ovarian origin, such as inhibin B and anti-mullerian hormone (AMH), may be useful to determine the follicular reserve when POF is suspected. Low levels of inhibin B may predict follicular depletion before the large FSH rise.

Karyotype evaluation and other cytogenetic investigations are useful to identify major X chromosome abnormalities.

## Differential diagnosis

The differential diagnosis is based on the exclusion of other causes of primary and secondary amenorrhea (absence of menstruation for more than 6 months). Parameters useful for the exclusion of each of the following conditions are illustrated:

• Pregnancy: high chorionic gonadotropin (CG) levels.

• Iatrogenic causes (surgery, anti-neoplastic treatments, radiations, antidopaminergic drugs): complete anamnestic investigation.

• Hypothalamic-pituitary disease (pituitary tumors, hyperprolactinemia, Kallmann syndrome, ....): high prolactin (PRL) and low/normal gonadotropin levels, alterations at imaging of brain/sella region.

• Hypothalamic amenorrhea (induced by stress, intensive exercise, anorexia, weight loss, fasting, severe diseases,): low/normal gonadotropin levels.

• Polycystic ovaries: alterations at ovarian ultrasound, normal gonadotropin and high androgen levels.

• Enzymatic defects of steroidogenesis (e.g. 21-hydroxylase deficiency): alterations at physical and adrenal ultrasound, normal gonadotropin, high androgen and adrenocorticotropic hormone (ACTH) levels.

• Endocrine disorders, such as hyperthyroidism, hypothyroidism, Cushing syndrome: complete clinical/biochemical evaluations, normal gonadotropin levels.

• Only in patients with primary amenorrhea:

- vaginal/uterus anatomical abnormalities, such as Rokitanski syndrome or Asherman syndrome: alterations at physical examination/pelvic ultrasound, normal gonadotropin levels.

- disorders of sexual differentiation (*e.g. *resistance to androgens): alterations at physical/ultrasound examination, evaluation of karyotype, measure androgen/anti-mullerian hormone levels.

## Genetic counseling

Genetic counseling is nowadays recommended for several reasons, when a genetic form of POF is suspected or identified.

Counseling is of particular importance in POF cases from families with X-linked mental retardation (Fragile X syndrome). Fragile X syndrome is due to CGG expansion (>55 repeats) at the 5'UTR of *FMR1 *gene (Xq27.3). The expansion of CGG repeats is associated with gene silencing resulting in male mental retardation and in POF with secondary amenorrhea in female carriers [8].

Genetic investigations may be useful for the early diagnosis of genetic defects underlying POF, when a female is born from a family with other female members affected with POF. Pedigree studies on affected families showed a mode of inheritance suggestive of autosomal dominant sex-limited transmission or X-linked inheritance with incomplete penetrance. In families with POF, the risk of other females developing POF will depend on the mode of inheritance and the mode of transmission. With autosomal dominant inheritance, the risk of POF will be 50% with either maternal or paternal transmission. However, with X-linked inheritance and paternal transmission this risk may be as high as 100%. These risks will be smaller with incomplete penetrance. If a POF patient appears to be a sporadic case, the risk of other female relatives developing POF will probably be equal to the risk in the general population.

All women who experience POF before the age of 30 years should perform a blood test for chromosomal assessment. Older women should discuss the option of chromosomal studies, as identification of abnormality may influence other family members, sisters or daughters, who carry the same defect in term of planning pregnancies. Carriers of the genetic defect may be advised for early pregnancy or oocyte collection and preservation.

## Antenatal diagnosis

Not relevant at present.

## Management

Patients with POF have infertility and hormone deficits. At present, fertility cannot be restored if the diagnosis is made after complete follicular depletion. In some cases, early diagnosis by genetic investigation may instead lead to advice for early conception or oocyte harvesting and preservation. Hormone defect may be substituted by estrogen/progestin preparations. The only solution presently available for the fertility defect in women with absent follicular reserve is represented by ovum donation.

## Unresolved questions

In most of the isolated defects the cause is still unknown. Several candidate genes have been identified, but causative mutations have been found in a strict minority of patients [7,11,15,16]. The prevalence of some genetic defects remains to be determined (*e.g. BMP15 *mutations). Though one paper described auto-antibodies against FSHR in a series of women with POF [24], auto-antigens and specific auto-antibodies for the diagnosis of autoimmune forms of isolated POF remain to be determined.
